# 

**Published:** 1842-03

**Authors:** 


### CONTENTS.

### LIBRARY.

PAGE.

Professor Baume's Work on First Dentition, translated by Thomas E. Bond, Jr. M. D. .......... 137

Principles of Dental Surgery; exhibiting a new method of treating the Diseases of the Teeth and Gums. By Leonard Koecker, Surgeon Dentist, London, .......... 1

### JOURNAL.

ARTICLE I.—Of Conjoined Suppuration of the Gums and Alveoli. By H. H. Hayden, M. D., of Baltimore, .......... 214

ART. II.—A Treatise on Mechanical Dentistry. By Solyman Brown, M. D., D. D. S. .......... 231

ART. III.—The use of Arsenious Acids for destroying the Nerve in Decayed Teeth. By W. E. Ide, M. D. of Zanesville, Ohio, ... 247

ART. IV.—Removal of the Superior Maxillary Bone, .......... 251

ART. V.—On the Vascularity of Dental Bone. By Chapin A. Harris, M. D., D. D. S. .......... 252

ART. VI.—Observations on Muco-purulent Secretion of the Antrum Maxillare. By S. P. Hullihen, Wheeling, Va. .......... 253

### MISCELLANEOUS NOTICES.

Bibliography, .......... 256

American Society of Dental Surgeons, .......... 256

Porcelain Teeth, .......... 258

Wonderful Discovery, .......... 259

Too wise to learn from books, .......... 260

Anchylosis of the Lower Maxilla with the Temporal Bone, .......... 260

Annual Commencement of Baltimore College of Dental Surgery, 261

Clerical Encouragement of Quackery, .......... 262

Dental and Surgical Instruments, .......... 263

Philadelphia Agent, .......... 263

Acknowledgment, .......... 263

Diseases of the Gums, .......... 263

Answers to Questions of a Correspondent, .......... 264

Married, .......... 264

Obituary, .......... 264

